# Busting the myth of extended blastocyst culture until Day 7: Protocol for systematic review and meta-analysis

**DOI:** 10.1097/MD.0000000000018909

**Published:** 2020-01-31

**Authors:** Alessandra Alteri, Laura Corti, Greta Chiara Cermisoni, Enrico Papaleo, Paola Viganò, Marco Noventa

**Affiliations:** a Obstetrics and Gynaecology Department, IRCCS San Raffaele Scientific Institute; b Reproductive Sciences Laboratory, Division of Genetics and Cell Biology, IRCCS San Raffaele Scientific Institute, Milan; c Department of Woman and Child Health, University of Padua, Padua, Italy.

**Keywords:** assisted reproductive technology, blastocyst, clinical pregnancy rate, Day 7 blastocyst, frozen embryo transfer, ongoing pregnancy rate

## Abstract

**Background:**

The prolonged culture of embryos to the blastocyst stage represents an increasing procedure in Assisted Reproductive Technology (ART) laboratories. Generally, only blastocysts developing on Day 5 and Day 6 are considered suitable embryos for transfer, cryopreservation or biopsy while embryos developed at a slower rate after Day 6 are routinely discarded. However, also blastocysts developing on Day 7 can be viable and result in a healthy live birth. Unfortunately, data regarding the clinical outcomes of Day 7 blastocysts compared to blastocysts developing on Day 5 or Day 6 are controversial. In this systematic review and aggregate data meta-analysis, we aim to evaluate the real reproductive potential of delayed blastocysts on Day 7 in frozen cycles.

**Methods:**

We will include all studies, with no restriction regarding the study design (randomized and observational trials, including cohort and case-control), investigating the clinical success of blastocysts developed on Day 7 compared to Day 5 and Day 6 blastocysts. The primary outcomes are the clinical pregnancy rate (CPR) and ongoing pregnancy rate (OPR) following frozen-thawed embryo transfer (Day 7 vs Day 6, and Day 5); secondary outcomes are: live birth rate (LBR) following frozen-thawed embryo transfer, euploid rate and survival rate of thawed blastocyst. Two reviewers independently will judge the methodological quality of studies included in the meta-analysis using the criteria reported in the Cochrane Handbook for Systematic Reviews of Interventions or the Newcastle-Ottawa Scale according to the design of the trials. The meta-analysis will be performed using random effects models and heterogeneity will be assessed using Higgins I2 value. Summary estimate of the proportion of each outcome will be expressed as pooled proportion with 95% confidence interval (CI). The effect of the day on each outcome will be evaluated using a multilevel mixed-effects model with a moderator (the day). The effect will be expressed as odds ratio (OR) with 95% confidence interval (CI). A *P* value less than .05 will be considered statistically significant.

**Ethics and dissemination:**

This is a systematic review not requiring an ethical approval. Findings derived from this systematic review and meta-analysis will be published in a peer-reviewed journal.

## 1 Introduction

Extended embryo culture to the blastocyst stage should represent the gold standard in Assisted Reproductive Technology (ART) cycles, improving embryo selection and encouraging single embryo transfer. A panel of experts defined that the optimal assessment of an embryo at blastocyst stage should be at 116 ± 2 h post insemination.^[1]^ Subsequently, the Vienna consensus considered the blastocyst development rate as a Key Performance Indicator, taking into account only blastocysts developed on Day 5.^[2]^ Moreover, this document declared that a possible additional Performance Indicator might be the development of an additional 10% to 15% of blastocysts within 140 ± 2 h post-insemination (blastocysts developed on Day 6).^[2]^ On the other hand, according to the American Society for Reproductive Medicine committee opinion document for blastocyst culture and transfer, current laboratory practice generally allows blastocyst cryopreservation both on Day 5 or Day 6.^[3]^ Very recently, a meta-analysis of retrospective observational studies regarding the outcomes of Day 5 vs Day 6 blastocyst transfers concluded that Day 5 blastocysts were associated with better clinical outcomes than Day 6 blastocysts in frozen cycles, suggesting a decrease of implantation potential of delayed Day 6 blastocysts.^[4]^

Much less attention has been dedicated over the years to the implantation potential of Day 7 blastocysts.^[5]^ Although their development is delayed compared to Day 5 or Day 6 blastocysts, Day 7 blastocysts can be viable, euploid, with a top morphological grade and can result in a healthy live birth.^[5]^ Data on euploid frequency, implantation, clinical pregnancy and live birth outcomes are anyway very controversial. Some studies showed that euploid rates of Day 7 blastocysts are comparable to those of Day 6 blastocysts,^[6–8]^ whereas others found that aneuploidy rate raised increasing timing of blastulation.^[9,10]^ Similarly, the implantation potential of Day 7 euploid blastocysts was not consistent among the studies.^[8–10]^ However, even if Day 7 euploid blastocyst transfers are associated to lower live birth rates compared to Day 5/Day 6 blastocysts,^[8,10]^ extended culture up to Day 7 should be considered in cases where an embryo is not available for biopsy on previous days.

Considering these controversies, we planned to perform a systematic review and meta-analysis to analyze the clinical outcomes after frozen transfer of blastocysts developing on Day 7 compared to those developed on Day 5/Day 6 in order to clarify the real reproductive potential of a thawed Day 7 blastocyst.

## 2 Methods

### 2.1 Protocol and registration

**Figure 1:**
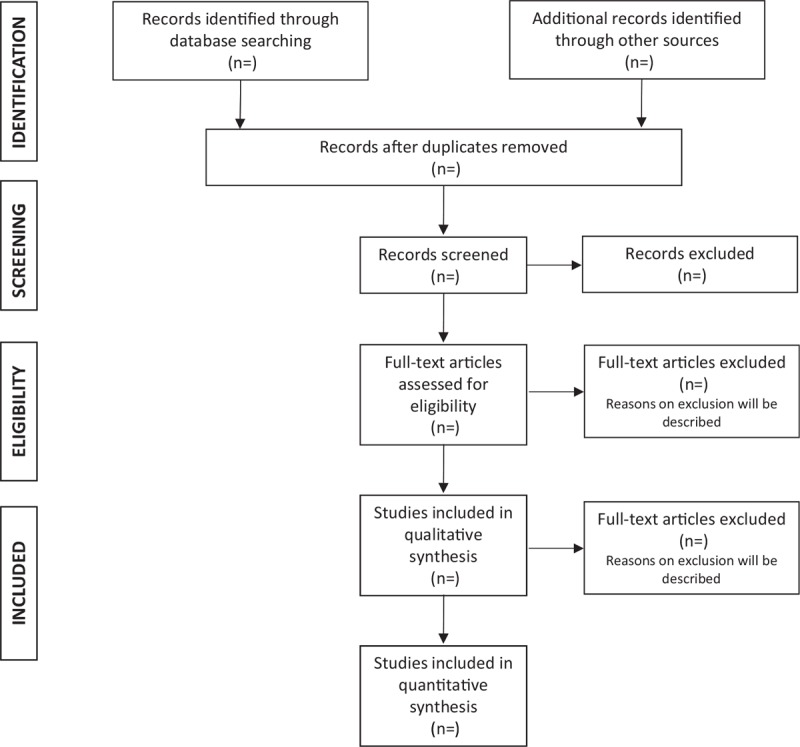
The PRISMA flow diagram for article selection and screening.

This protocol for systematic review was registered to the International Prospective Register of Systematic Reviews (PROSPERO)^[11]^ with the registration number CRD42019129502. The Preferred Reporting Items for Systematic Reviews and Meta-Analyses (PRISMA-P) guidelines were used to design this systematic review protocol.^[12]^ We will include a proposed flow diagram that shows the search process (Fig. 1).

### 2.2 Search strategy

We will search the following electronic bibliographic databases: PubMed, Scopus, EMBASE, The Cochrane Library (Cochrane Database of Systematic Reviews, and Cochrane Central Register of Controlled Trials (CENTRAL), Cochrane Methodology Register). The search strategy will include only terms relating to or describing the intervention. The search terms will be adapted for use with other bibliographic databases in combination with database-specific filters for controlled trials, where these are available. We will include only manuscripts published in English language.

The key search terms will include: (EMBRYO OR embryos OR BLASTOCYST∗) AND (“day 7” OR day-7 OR day7 OR “blastocyst development” OR “morphokinetic” OR “blastocyst morphology”) AND (transfer∗ OR euploid∗ OR aneuploid∗ OR implantation OR pregnancy OR live birth).

### 2.3 Eligibility criteria

#### 2.3.1 Study design

We will include all studies reported in English language with no restriction regarding the study design (randomized and observational trials, including cohort and case-control), published from inception to August 2019. Included manuscripts must report data about live birth rate (LBR), clinical pregnancy rate (CPR), ongoing pregnancy rate (OPR) and euploid rate comparing different stages of blastocyst development (Day 7, Day 6, and Day 5).

#### 2.3.2 Population

The study will include infertile patients undergoing frozen/thawed blastocyst transfers in ART cycles. A subgroup analysis according to patients’ characteristics (e.g., PGT-A or donor cycles) is also planned.

### 2.4 Intervention and comparisons

#### 2.4.1 Outcomes

The primary outcomes are the CPR and OPR following frozen-thawed embryo transfer. CPR is defined as the presence of at least one intrauterine gestational sac with viable fetus while OPR is defined as the number of pregnancies confirmed by ultrasound scan and continued for at least 21 weeks after embryo transfer. Secondary outcomes are euploid rate, LBR following frozen-thawed embryo transfer and survival rate of thawed blastocyst.

#### 2.4.2 Exposures of interest

Exposed/intervention group will consist of patients with blastocysts cryopreserved on Day 7 in freeze-all cycles. As control group, we will include patients with blastocysts cryopreserved on Day 5 or Day 6 in freeze-all cycles.

### 2.5 Patient and public involvement

Patients were not involved in the development of this study protocol. This study will use publically available data without patient's identification.

### 2.6 Study selection and data extraction process

Titles and/or abstracts of studies retrieved using the search strategy and those from additional sources will be screened independently by two reviewers to identify studies that potentially meet the inclusion criteria outlined above. The full texts of these potentially eligible studies will then be retrieved and independently assessed for eligibility, again by two review team members. Any disagreements over the eligibility of studies will be resolved through discussion with a third reviewer. A standardized, pre-piloted form will be used to extract data from the included studies for assessment of study quality and evidence synthesis. Extracted information will include: study setting; study population and baseline characteristics; details of the intervention and control conditions; study methodology and information for the assessment of the risk of bias. Any missing data will be requested from the study authors.

### 2.7 Risk of bias in included studies

Two reviewers independently will judge the methodological quality of studies included in the meta-analysis.

In case of randomized trials, we will apply the criteria reported in the Cochrane Handbook for Systematic Reviews of Interventions. In particular, seven specific domains related to risk of bias will be assessed: random sequence generation; allocation concealment; blinding of participants and personnel; blinding of outcome assessment; incomplete outcome data; selective data reporting; other bias. In case of non-randomized trials, we will apply the Newcastle-Ottawa Scale. In particular, three specific domains related to risk of bias will be assessed: selection, comparability, and outcome. Authors’ judgments will express as “low risk”, “high risk” or “unclear risk” of bias. Disagreements between the review authors over the risk of bias will be resolved by discussion, with involvement of a third review author.

### 2.8 Data synthesis

Titles and abstracts will be independently screened by 2 authors. The same authors independently will assess studies for inclusion, check the reference lists of retrieved studies and assess the methodological quality of included studies. The same two authors independently will collect data both for qualitative and quantitative analysis using a pre-piloted form. After this process, all collected data for each manuscript will be discussed together with a third reviewer. Any discrepancy will be discussed and resolved reanalyzing together each single manuscript.

According to Cochrane statement (considering that this is the first systematic review with meta-analysis on this topic) we plan to perform an aggregate data meta-analysis. In case of insufficient information in full-text papers, we will contact authors (by e-mail) to ask for additional data.

The study will consist of two major sections. In the first one, we will provide a qualitative synthesis (systematic review) of the findings. In particular, for each study, we will analyze the design of the trials, the general features of included population, the insemination methods, the number of retrieved oocytes, the number of obtained blastocyst (according to the days of blastocyst development), the embryo quality, the euploid rate, the number of embryo transfer cycles, the CPR per embryo transfer and the LBR per embryo transfer. Euploid rate, CPR and LBR will be evaluated according to the day of blastocyst development (Day 5, Day 6, and Day 7).

In the second section we will provide a meta-analysis of included papers according to the previous cited outcomes. The pooled results of CPR, euploid rate, survival rate of thawed blastocysts and LBR will be compared according to the day of blastocyst development (Day 5, Day 6, and Day 7).

### 2.9 Statistical analysis

Statistical analysis will be performed using “metafor” package for R 3.5 (R Foundation for Statistical Computing, Vienna, Austria). Meta-analysis will be performed using random effects models and heterogeneity will be assessed using Higgins I2 value. Summary estimate of the proportion of each outcome will be expressed as pooled proportion with 95% confidence interval (CI). The effect of the day on each outcome will be evaluated using a multilevel mixed-effects model with a moderator (the day). The effect will be expressed as odds ratio (OR) with 95% confidence interval (CI). A *P* value less than .05 will be considered statistically significant. If possible, sources of heterogeneity will be explored by with sensitivity analysis (by excluding each study and different study subgroups [according to the risk of bias scores] from aggregate analysis) and subgroup analysis.

### 2.10 Ethics and dissemination

Ethical approval is not required because this systematic review will use the published data. Findings of this systematic review will be presented at relevant conferences and published in a peer-reviewed journal.

## 3 Discussion

Implementation of extended blastocyst culture to Day 7 is not a widely accepted practice in the IVF laboratories. Data from literature suggested that some of slower Day 7 blastocysts may be euploid and result in a live birth but the results of these studies seem to be controversial. To clarify the implantation potential of a Day 7 blastocyst, a systematic review and meta-analysis will be performed. A study of this type will clarify whether the extended culture to Day 7 represents an extra chance for those patients who do not have useable embryos for cryopreservation and/or biopsy on Day 5 or Day 6. The clinical practice might possibly benefit from the deriving observations.

## Author contributions

**Conceptualization:** Alessandra Alteri, Laura Corti, Greta Chiara Cermisoni.

**Funding acquisition:** Alessandra Alteri.

**Investigation:** Paola Viganò, Marco Noventa.

**Methodology:** Marco Noventa.

**Resources:** Laura Corti.

**Supervision:** Enrico Papaleo, Paola Viganò, Marco Noventa.

**Validation:** Marco Noventa.

**Writing – original draft:** Alessandra Alteri.

**Writing – review & editing:** Alessandra Alteri, Laura Corti, Greta Chiara Cermisoni, Paola Viganò, Marco Noventa.

Alessandra Alteri orcid: 0000-0003-3092-3546.
